# Study on the Pattern of Postpartum Uterine Involution in Dairy Cows

**DOI:** 10.3390/ani13233693

**Published:** 2023-11-29

**Authors:** Tianshu Dai, Ziming Ma, Xingru Guo, Shihao Wei, Baolong Ding, Yun Ma, Xingang Dan

**Affiliations:** College of Animal Science and Technology, Ningxia University, Yinchuan 750021, China; daitianshu@stu.nxu.edu.cn (T.D.); hnpdsmzm@163.com (Z.M.); fakegxr1999@163.com (X.G.); 12020131336@stu.nxu.edu.cn (S.W.); ding113610@163.com (B.D.); tmlf74@126.com (Y.M.)

**Keywords:** dairy cows, E2, hydroxyproline, IGF-1, uterine involution

## Abstract

**Simple Summary:**

Postpartum uterine involution is necessary for the normal reproduction of dairy cows. The study aimed to investigate the pattern of postpartum uterine involution and the effect of parity on uterine involution in dairy cows. A B-mode veterinary ultrasound scanner was used to monitor the diameter of the uterine cervix, pregnant uterine horn, and non-pregnant uterine horn at 5, 10, 15, 20, 25, 30, 35, and 40 days, respectively, after parturition in both multiparous and primiparous dairy cows. Meanwhile, the concentrations of hydroxyproline, estradiol (E2), and insulin-like growth factor 1 (IGF-1) were detected using ELISA and blood samples. The results showed that the diameter of the pregnant uterine horn and uterine cervix did not decrease any further at 25 days postpartum. Hydroxyproline levels gradually decreased during uterine involution; however, there was no significant variation in IGF-1 and E2 concentrations during uterine involution. These results suggest that uterine involution was around 25 days postpartum in healthy dairy cows. The hydroxyproline levels of the peripheral blood may be an indicator of uterine involution in postpartum cows.

**Abstract:**

Postpartum uterine involution is necessary for the normal reproduction of dairy cows. The study aimed to investigate the pattern of postpartum uterine involution and the impact of parity on uterine involution in Chinese Holstein dairy cows. The diameter of the uterine cervix, pregnant uterine horn, and non-pregnant uterine horn were monitored using a B-mode veterinary ultrasound scanner at 5, 10, 15, 20, 25, 30, 35, and 40 days, respectively, after parturition in both multiparous and primiparous dairy cows. Meanwhile, the concentrations of hydroxyproline, E2, and IGF-1 were detected using ELISA at 5, 10, 15, 20, 30, and 40 d after parturition in both multiparous and primiparous dairy cows. Furthermore, the duration of uterine involution was compared in the multiparous and primiparous dairy cows. The results demonstrated that the diameter of the uterine cervix and the pregnant uterine horn did not decrease any further at 25 days postpartum for both the multiparous cows and the primiparous cows. Hydroxyproline levels gradually decreased with uterine involution; however, there was no significant variation in IGF-1 concentrations during uterine involution in the dairy cows. Although E2 concentrations of the peripheral plasma displayed an upward trend from day 5 to day 15 in the two groups of postpartum cows, there was no significant difference between the two groups during uterine involution. These results suggest that postpartum uterine involution was around 25 days postpartum in both the primiparous dairy cows and the multiparous Chinese Holstein dairy cows. Parity did not affect uterine involution in the postpartum Chinese Holstein dairy cows. The hydroxyproline levels of the peripheral blood may be an indicator of uterine involution in postpartum cows. Nonetheless, IGF-1 and E2 levels of the periphery blood are not associated with uterine involution in Chinese Holstein dairy cows.

## 1. Introduction

Uterine involution is a physiological process through which the uterus turns to its pre-pregnancy dimensions with endometrial regeneration, reduced uterine blood flow, endometrial vascularity, and reduced muscle mass. The uterus of dairy cows undergoes morphological, structural, and functional changes after parturition, subsequently restoring to its pre-pregnancy status, which is important to ensure the initiation of the oestrus cycle and the normal reproduction of postpartum cows [1]. Furthermore, healthy uterine involution is a prerequisite for artificial insemination of dairy cows and is crucial for postpartum cows to conceive again [2]. In healthy postpartum cows, when the size of the cervix and uterine horn does not change any further, this indicates that uterine involution is almost complete. At this point, estrous synchronization and fixed-time artificial insemination are implemented in healthy cows with uterine involution, which shortens the calving interval and further improves the reproductive efficiency of dairy cows. Studies have reported that parity, nutritional conditions, body condition score at calving, and postpartum uterine disease, including metritis, endometritis hyperketonemia, lipomobilization, and hypocalcemia, delayed postpartum uterine involution in dairy cows [3,4,5,6,7]. This increases the calving interval and significantly reduces the reproductive rate of dairy cows. However, the results of studies on the effect of parity affecting uterine involution are inconsistent. A previous study demonstrated that uterine involution of multiparous dairy cows was shorter than that of primiparous dairy cows [8]. However, another study indicated that the parity of Finnish dairy cows exhibited no significant impact on the recovery of the uterus or cervix in postpartum cows [9]. Thus, further investigations of the uterus, cervix, and horn are important to validate whether parity influences uterine involution.

In multiple species, ultrasound has proven to be the most practical and accurate tool for monitoring uterine involution [10]. The length and width of a uterus can be measured in humans using 2D or 3D ultrasound [11,12,13]. The B-mode ultrasound scanner is extensively used for pregnancy diagnosis in animals and for assessing uterine involution in postpartum cows [14,15]. In addition, it was reported that the process of uterine involution is accompanied by tissue collagen degradation and produces free glycine and hydroxyproline in the blood, which is closely associated with uterine involution [16]. The process of postpartum uterine involution not only involves the degradation of uterine collagen fiber but also involves the repair and regeneration of the endometrium in dairy cows. It was reported that IGFs stimulate the proliferation of postpartum endometrial cells via the PI3K/AKT/mTOR pathway, which in turn promotes endometrial repair and regeneration in dairy cows [17]. It was further observed that low levels of IGF-1 delayed postpartum uterine involution in dairy cows [18]. In addition, E2 also indirectly modulated the proliferation of endometrial cells by up-regulating the IGF-1 in uterine tissue [19]. However, to date, studies on E2 and IGF-1-based regulation of uterine involution in postpartum dairy cows are limited, and whether E2 and IGF-1 concentrations change with uterine involution remains elusive.

Therefore, to clarify the effect of parity on uterine involution in postpartum dairy cows, the diameters of the uterine cervix, pregnant uterine horn, and non-pregnant uterine horn during uterine involution of healthy multiparous and primiparous dairy cows were monitored using B-mode ultrasonography, respectively. Meanwhile, the levels of hydroxyproline, IGF-1, and E2 in the peripheral blood were respectively determined during uterine involution. Our study will clarify the time point of uterine involution in postpartum healthy Holstein cows and the correlation between the diameters of the cervical and pregnant uterine horn with the number of postpartum days. Additionally, our study will also confirm whether parity, IGF-1, and E2 of the peripheral blood are associated with uterine involution. This could reveal the pattern of uterine involution in healthy postpartum cows, which may provide some reference for estrous synchronization and fixed-time artificial insemination of postpartum dairy cows on farms.

## 2. Materials and Methods

### 2.1. Animals

The study was conducted on a 10,000-head dairy farm in the Ningxia region between August 2022 and October 2022. In total, 32 primiparous Chinese Holstein dairy cows calving on the same day were selected for this experiment. Forty days later, 32 multiparous Chinese Holstein dairy cows (third calving) were also selected to complete the follow-up experiment. Some dairy cows with metritis, endometritis periparturient diseases, calving difficulty, and other postpartum diseases were eliminated during the experiment, leaving only healthy dairy cows for subsequent experiments. All experimental techniques were performed per the guidelines of the Committee of Animal Research Institute, Ningxia University, China. The dairy cows received an adequate amount of total mixed ration per day to cover their energy needs and sustain BCS.

### 2.2. Ultrasonographic Detection

A Gandalf B-mode veterinary ultrasound machine (Gandalf, Zhengzhou, China, GDF-C70) with an 8.0 MHz electronic inverter line array probe for ultrasound monitoring was used in the experiment. The transducer coated with lubricant gel and protected with a lubricated plastic obstetrical sleeve was inserted and guided to the required position after the manual evacuation of feces from the rectum. Cross-sectional images were obtained by appropriately positioning the transducer. Cervical diameters were measured by placing the transducer over the middle of the cervix. To determine the uterine horn diameters, the transducer was placed 10 cm cranially to the bifurcation of the uterus. The distance from serosa to serosa was considered to correspond to the cervical and uterine horn diameters. Ultrasonographic detection was completed by a skilled veterinarian during the entire experiment. The cervical diameters for all experimental dairy cows were measured using the electronic scale of ultrasound at 5, 10, 15, 20, 25, 30, 35, and 40 days, respectively, after parturition. Meanwhile, the diameters of the pregnant uterine horn and non-pregnant uterine horn for all experimental dairy cows were determined with the electronic scale of ultrasound at 15, 20, 25, 30, 35, and 40 days after parturition.

### 2.3. Collection of Blood Samples

Blood samples were collected from all the experimental dairy cows at 5, 10, 15, 20, 30, and 40 days postpartum through the tail vein, placed in tubes containing EDTA, centrifuged at 4000 rpm for 8 min. Subsequently, the supernatant was aspirated in clean tubes and stored in a −80 °C refrigerator. Hydroxyproline, E2, and IGF-1 levels in blood were determined using ELISA.

### 2.4. ELISA Detection

Bovine hydroxyproline ELISA kit YJ665869 (Mibio, Shanghai, China) with CV% < 15% between the intra- and inter-assay was used to detect the concentration of hydroxyproline in blood samples. Bovine E2 ELISA kit CSB-E08173b (Cusabio, Wuhan, China) with CV < 15% between the intra- and inter-assay was used to detect the concentration of estradiol in blood samples. Bovine IGF-1 ELISA kit EB6RB (Invitrogen, Carlsbad, CA, USA) was used to determine the concentration of IGF-1 in blood samples. The intra- and inter-assay coefficients of variation for IGF-1 were less than 10% and 12%, respectively. The detection ranges of hydroxyproline, E2, and IGF-1 assay kits were 0.2–30 µg/mL, 50–1200 pg/mL, and 1.23–300 ng/mL, respectively. ELISA detection was conducted according to the kit’s instructions.

### 2.5. Statistical Analysis

Experimental data were presented as mean ± SD. The one-way analysis of variance was used to determine the statistically significant difference among the experimental groups using IBM SPSS 21.0 software (SPSS Inc., Chicago, IL, USA) and using post-hoc Tukey’s multiple comparisons. *p* < 0.05 was considered statistically significant. For regression analysis, the diameters of the cervix and pregnant uterine horn and the HPY levels were regarded as dependent variables and postpartum days as independent variables. The equation of linear regression was obtained based on the highest correlation coefficient (R2).

## 3. Results

### 3.1. Changing Patterns of Cervix during Postpartum Uterine Involution in Dairy Cows

**Figure 1:**
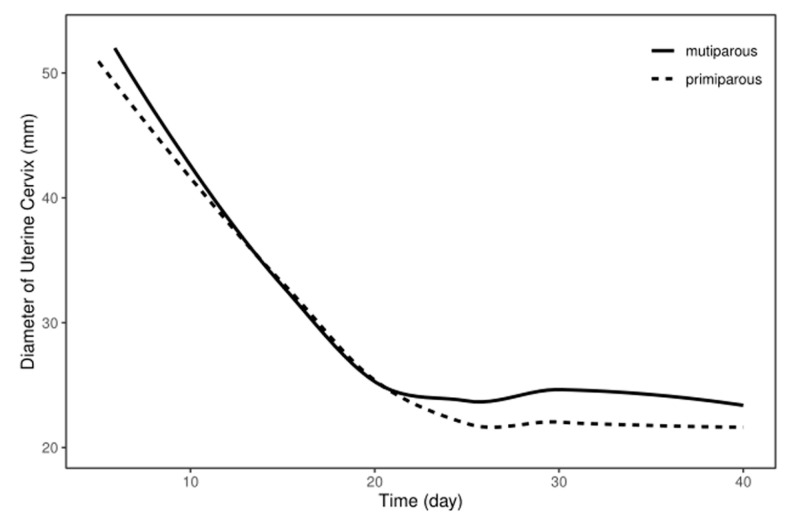
The involution pattern of cervix in postpartum Holstein dairy cows. Cervix diameters gradually declined after calving in both multiparous and primiparous dairy cows.

We monitored the cervical alterations at various points after parturition in both multiparous and primiparous dairy cows (Figures S1 and S2) and performed a statistical analysis of cervical diameter in both groups of dairy cows. The cervical diameter decreased significantly at 5, 10, 15, and 20 days in the multiparous dairy cows (*p* < 0.05), whereas there was no significant difference observed in cervical diameter between day 20 and day 40 postpartum (Table 1). Additionally, the cervical diameter decreased significantly at 5, 10, 15, and 20 days in the primiparous dairy cows (*p* < 0.05), whereas there was no significant difference observed in cervical diameter between day 25 and day 40 postpartum (Table 1). Finally, regression curves were analyzed to show the pattern of the cervix in the postpartum multiparous and primiparous dairy cows (Figure 1). The regression model for the correlation between the reduced diameter of the cervix and the number of postpartum days revealed the quartic curve as the highest correlation coefficient. The R^2^ for the multiparous and primiparous cows was 0.9128 and 0.9595, respectively. This suggests that recovery of the postpartum cervix takes 20 days postpartum in the multiparous dairy cows, while recovery of the postpartum cervix in the primiparous dairy cows occurs 25 days postpartum.

### 3.2. Changing Patterns of Pregnant Uterine Horn and Non-Pregnant Uterine Horn

**Figure 2:**
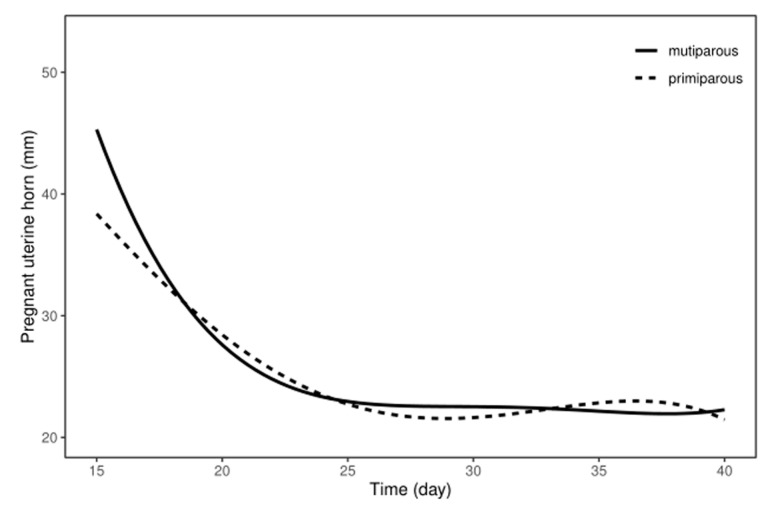
The involution pattern of pregnant uterine horn in postpartum Holstein dairy cows. Pregnant uterine horn diameters gradually declined after calving in both multiparous and primiparous dairy cows.

We performed a B-mode ultrasound examination of the pregnant uterine horn and non-pregnant uterine horn during uterine involution in both the multiparous and the primiparous dairy cows (S3–S6). The diameters of the pregnant uterine horn and non-pregnant uterine horn were, respectively, analyzed during uterine involution. The results showed that the diameter of the pregnant uterine horn decreased significantly from day 15 to day 25 postpartum in both the multiparous and the primiparous dairy cows (*p* < 0.05), whereas there was no significant difference observed between day 25 and day 40 postpartum (Table 2). Additionally, regression curves were analyzed to show the pattern of the pregnant uterine horn in the postpartum multiparous and primiparous dairy cows (Figure 2). The regression model for the correlation between the reduced diameter of the pregnant uterine horn and the number of postpartum days revealed the quartic curve as having the highest correlation coefficient. The R^2^ for the multiparous and primiparous was 0.9314 and 0.9267, respectively. This suggests that recovery of the pregnant uterine horn can be achieved at 25 days postpartum in both multiparous dairy cows and primiparous dairy cows. In addition, no significant changes in non-pregnant uterine horn diameter in either multiparous dairy cows or primiparous dairy cows were observed throughout uterine involution (Table 2).

### 3.3. Changes in Blood Levels of Hydroxyproline, IGF-1, and E2 in Dairy Cows at Different Time Points after Parturition

**Figure 3:**
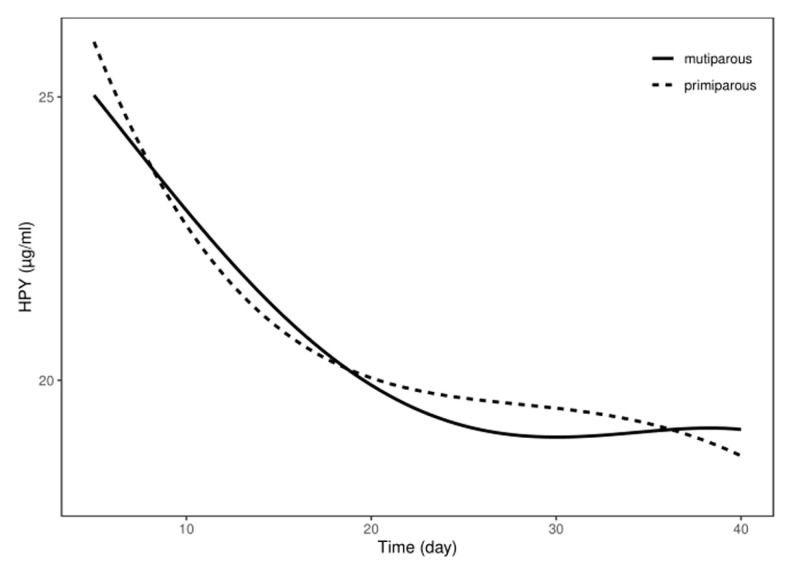
The concentration changes of HPY in postpartum Holstein dairy cows. HPY levels in peripheral blood gradually declined after calving in both multiparous and primiparous dairy cows.

**Figure 4:**
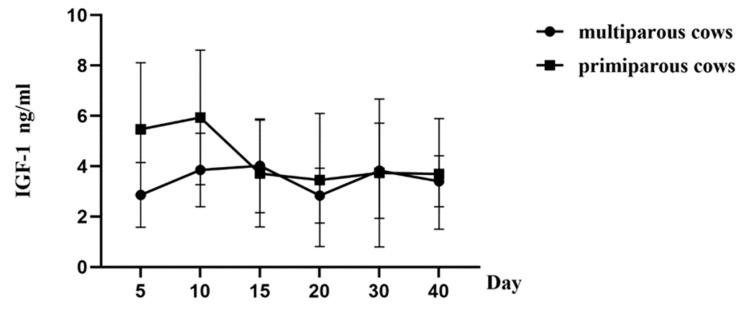
IGF-1 levels change in the blood of multiparous and primiparous dairy cows at different times after parturition. Cows were assigned into either multiparous group (multiparous dairy cows, *n* = 32) or primiparous group (primiparous dairy cows, *n* = 32). The concentrations of Estradiol were detected by ELISA method at 5, 10, 15, 20, 30, and 40 days after parturition in both multiparous and primiparous dairy cows.

**Figure 5:**
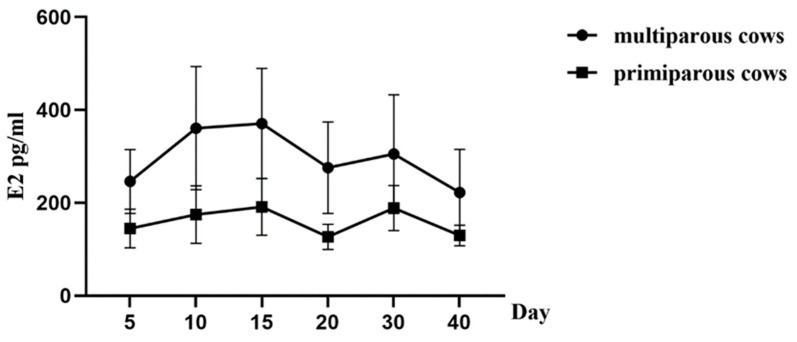
E2 levels changes in the blood of multiparous and primiparous dairy cows at different time points after parturition. Cows were assigned into either multiparous group (multiparous dairy cows, *n* = 32) or primiparous group (primiparous dairy cows, *n* = 32). The concentrations of insulin-like growth factor 1 were detected by ELISA method at 5, 10, 15, 20, 30, and 40 days, respectively, after parturition in both multiparous and primiparous dairy cows.

The hydroxyproline levels in the periphery blood decreased significantly from day 5 to day 10 postpartum in both the multiparous and the primiparous dairy cows (*p* < 0.05), whereas there was no significant difference observed between day 20 and day 40 postpartum in the two groups. Further, regression curves were analyzed to show the pattern of HPY levels in the postpartum multiparous and primiparous dairy cows (Figure 3). The regression model for the correlation between the reduced HPY concentrations and the number of postpartum days revealed the quartic curve as having the highest correlation coefficient. The R^2^ for the multiparous and the primiparous cows was 0.8972 and 0.8326, respectively. Furthermore, IGF-1 levels in plasma gradually increased 5–10 days after parturition in both the multiparous and the primiparous dairy cows, whereas the plasma IGF-1 levels showed no significant differences at different periods of uterine involution in both the multiparous and the primiparous dairy cows (Figure 4). No significant differences in IFG-1 levels were observed at 5, 10, 15, 20, 30, and 40 days postpartum between the multiparous and the primiparous dairy cows. Additionally, E2 concentrations of the peripheral blood tended to increase at 5–15 days postpartum in both the multiparous cows and the primiparous cows, whereas there were no significant differences observed at different periods of uterine involution in the multiparous and the primiparous dairy cows (Figure 5). This suggests that HPY levels may be used as an indicator of uterine involution, whereas IGF-1 and E2 levels were not associated with uterine involution in the dairy cows.

## 4. Discussion

In dairy cows, postpartum uterine involution is a complex process involving the recovery of the uterus, degeneration of the myometrium, and regeneration of the endometrium, which is essential for them to become pregnant again. A comprehensive understanding of the pattern of uterine involution is required for the reproductive function of dairy cows after parturition. During uterine involution, a major characteristic of the bovine uterus is the contraction of uterus size [20]. From the first day after parturition, the uterus gradually restores to its pre-pregnancy size with the contraction of the uterine muscles and the discharge of the effluvia [6]. During this period, the changes in the cervix and uterine horns can reflect the status of uterine involution. In this study, we observed that the time point of postpartum cervix recovery was 20 days postpartum in the multiparous dairy cows and 25 days postpartum in the primiparous dairy cows. The recovery time of the postpartum pregnant uterine horn is 25 days after parturition in both the multiparous and primiparous dairy cows. A previous study has indicated that cervical involution was completed at 30.1 ± 3.8 days in first-calving cows and 30 ± 4.3 days in third-calving cows [9]. This is consistent with our results. In addition, Heppelmann et al. [20] observed that the reduction in diameter of the pregnant uterine horn of healthy cows tended to level off after day 25 postpartum. Furthermore, Zhang et al. [4] indicated that the decline in diameter of the pregnant uterine horn tended to level off at day 25 postpartum in the second and multiparous Chinese Holstein cows. These are consistent with our results. Thus, it may be concluded that the time point of postpartum uterine involution is 25 days after parturition in both multiparous dairy cows and primiparous dairy cows.

Nonetheless, there is still controversy about the period of uterine involution between primiparous and multiparous dairy cows. A study has demonstrated that parity and calving age significantly impacted uterine involution in Chinese Holstein cows, and the recovery of the pregnant uterine horn in biparous and multiparous dairy cows was faster than that of the primiparous dairy cows during the uterine involution period [2]. In addition, another study has revealed a significant negative correlation between parity and postpartum uterine involution in Chinese Holstein cows, and that the time of postpartum pregnant uterine horn involution shortens with increasing parity [4]. However, in our study, the time for the recovery of the uterus was 25 days for both multiparous and primiparous dairy cows. This indicated that parity may not affect the length of time for uterus involution in dairy cows. A previous study has also revealed that the parity in Finnish cows exhibited no significant impact on uterine or cervical involution [9]. This is consistent with our results. Interestingly, we observed that the cervix recovery in the multiparous dairy cows was faster than that of the primiparous dairy cows. In contrast, Zhang et al. [4] indicated that there was no significant difference observed in the change of cervical diameter after calving among primiparous, biparous, and multiparous Chinese Holstein cows. However, the pregnant uterine horn recovery in biparous and multiparous cows was faster than that of primiparous dairy cows. The reason for the discrepancy remains elusive.

Collagen is a fibrous protein containing glycine, proline, and hydroxyproline. Collagen is involved both in placental development and in the uterine involution processes. The increase in uterine weight throughout pregnancy is accompanied by collagen deposition. The uterus exhibits a gradual increase in collagen content during pregnancy [21]. In particular, the collagen content of the uterus further increases in late gestation to support the increasing fetal load [22]. However, the size and weight of the uterus are reduced due to the rapid degradation of collagen after parturition [22]. After calving, the collagen is degraded by the action of collagenase culminating in the appearance of free glycine and hydroxyproline in the blood. Hydroxyproline is not present in feedstuffs and is unique to collagen [16]. Therefore, the concentration of hydroxyproline in the blood was correlated with uterine involution and regarded as an indicator of uterine involution [16]. In dairy cows, uterine involution was assessed by detecting the hydroxyproline levels in the periphery blood [23]. In this study, we determined that hydroxyproline levels were higher in the blood at 5–15 days postpartum, indicating that a large amount of collagen was degraded during this period and the uterine tissue was undergoing rapid recovery. We also observed that the diameter of the cervix decreased rapidly during this period. The cervix mainly comprises connective tissue and smooth muscle. The degradation of collagen in the muscle and connective tissue of the uterus was associated with uterine involution; thus, hydroxyproline was often considered to be an indicator of uterus tissue collagen degradation [24,25,26]. This suggests that the hydroxyproline level in the peripheral blood is correlated with cervical diameter, and the hydroxyproline levels also gradually decreased with the decline in cervical diameter. Overall, the hydroxyproline levels in the peripheral plasma reflect the metabolism of uterine collagen, which is positively correlated with the decline in cervical diameter. Therefore, the hydroxyproline levels in the peripheral blood may be used as an indicator for estimating postpartum uterine involution in dairy cows.

It was shown that IGF-1 may promote the proliferation of uterine stroma membrane and epithelial cells and collagen synthesis in a paracrine or autocrine manner during postpartum uterine involution in dairy cows [27,28]. Further, a study has reported that IGF-1 can bind to its receptor (IGF-1R) and enhance uterine cell proliferation by activating the PI3K/AKT and Ras/Raf/MAPK signaling pathways [29]. Additionally, another study also indicated that the insulin signaling pathways were altered in the endometrial epithelial cells of postpartum cows with a severe negative energy balance, which in turn affects the rate of endometrial repair [30]. In this study, we demonstrated that plasma concentrations of IGF-1 increased slightly at 5–10 days after parturition and further decreased. A previous study depicted that plasma IGF-1 concentrations decreased sharply in postpartum cows at 14 days prior to parturition and reached a minimum at 4 days postpartum, and subsequently remained low until day 9 postpartum [31]. This is consistent with our findings. IGF-1 is mainly synthesized in the liver, and the liver reduces IGF-1 production during the early lactation period for negative energy balance, malnutrition, disease, or inflammation, which in turn affects the concentration of IGF-1 in the blood [18,32,33,34]. In addition, Llewellyn et al. [27] indicated that mRNA for IGF-1 was highly expressed in endometrial tissue in the early postpartum period and promoted endometrial repair during uterine involution. However, our results indicated that the IGF-1 levels in the peripheral blood were not associated with postpartum uterine involution in dairy cows. We speculated that IGF-1 levels in the peripheral blood were not indicative of the concentration of IGF-1 synthesized by endometrial tissue during endometrial tissue regeneration; thus, it may not be used as an indicator of endometrial repair during uterine involution.

E2 is an important steroid hormone that plays a major role in female reproduction (e.g., follicle development, fertilization, pregnancy establishment, and maintenance) [35,36]. It was reported that an increase in P_4_ concentration led to an increase in *Zinc Finger E-Box Binding Homeobox 1* (*ZEB1*) concentrations, which in turn inhibits the expression of the contraction-related genes *Oxytocin Receptor*, *Connexin 43,* and *Cyclooxygenase 2*, and myometrial contraction [37]. Higher E2 concentrations inhibit *ZEB1* expression, up-regulate contraction-related genes, and promote myometrial contractions [37]. Our results have demonstrated that there was a gradual increase in E2 levels at 5–15 days postpartum, whereas the diameter of the cervix and uterine horns decreased rapidly during this period. Therefore, we speculated that high E2 levels in the early postpartum period promoted the contraction of the myometrium and the elimination of lochia, which caused the cervix and uterine horns to restore to their pre-pregnancy physiological position. In addition, E2 promotes the repair of uterine tissue after parturition. It increases blood flow to the postpartum myometrium, endometrium, and uterine sarcolemma, which provides abundant nutrients for the growth and repair of damaged uterine tissue in the postpartum period [38]. Furthermore, E2 mediated the mitogenesis of endometrial cells by increasing the expression of *IGF-1* in the uterus [19]. It also promotes endometrial regeneration by increasing the phosphorylation of PKB/AKT in endometrial cells [39]. Thus, high levels of E2 in the early postpartum period may contribute to uterine involution in dairy cows.

## 5. Conclusions

In the present study, the recovery time of uterine involution was 25 days in both multiparous and primiparous dairy cows. Parity did not impact uterine involution in postpartum Holstein cows. The hydroxyproline concentration in the periphery blood was positively correlated with uterine involution, which may be used as an indicator of uterine involution in dairy cows. The E2 and IGF-1 levels in the peripheral blood were not associated with uterine involution in postpartum dairy cows.

## Figures and Tables

**Table 1:** The diameters of cervix, pregnancy horn, and non-pregnancy horn at different periods of uterine involution in both multiparous and primiparous dairy cows (mm).

	Cervix of Multiparous Dairy Cows	Cervix of Primiparous Dairy Cows	Pregnancy Horns of Multiparous Dairy Cows	Pregnancy Horns of Primiparous Dairy Cows	Non-Pregnancy Horns of Multiparous Dairy Cows	Non-Pregnancy Horns of Primiparous Dairy Cows
5 d	53.85 ± 3.33 a	50.64 ± 3.44 a				
10 d	44.86 ± 8.51 b	41.93 ± 3.42 b				
15 d	36.82 ± 6.4 c	34.39 ± 5.38 c	42.64 ± 7.17 a	37.57 ± 5.15 a	23.98 ± 1.84 a	21.26 ± 2.1
20 d	25.29 ± 5.3 d	24.49 ± 5.37 d	28.88 ± 4.73 b	27.19 ± 5.12 b	20.51 ± 3.35 b	20.95 ± 3.33
25 d	24.53 ± 1.91 d	22.19 ± 2.48 e	22.62 ± 2.99 c	20.96 ± 2.76 c	19.91 ± 2.99 b	19.34 ± 2.25
30 d	25.08 ± 1.34 d	22.27 ± 2.21 ef	23.34 ± 3.26 c	22.33 ± 2.59 c	20.95 ± 2.28 b	19.13 ± 2.54
35 d	24.51 ± 2.61 d	22.19 ± 2.83 ef	22.42 ± 2.69 c	22.05 ± 2.48 c	20.4 ± 1.74 b	19.73 ± 2.26
40 d	22.16 ± 2.15 d	20.98 ± 2.3 ef	21.95 ± 1.38 c	19.96 ± 1.95 c	19.4 ± 2.71 b	18.5 ± 2.4

Note: Cows were assigned into either multiparous group (multiparous dairy cows, *n* = 32) or primiparous group (primiparous dairy cows, *n* = 32). Significant differences are indicated between completely different letters in the same vertical row, *p* < 0.05.

**Table 2:** HPY levels of peripheral blood at different periods of postpartum uterine involution in dairy cows.

	5 d	10 d	15 d	20 d	30 d	40 d
multiparous	25.083 ± 1.9 a	22.74 ± 2.65 b	21.625 ± 2.862 bc	19.656 ± 2.23 cd	19.039 ± 2.05 d	19.125 ± 2.038 d
primiparous	26.121 ± 3.66 a	22.442 ± 1.91 b	21.589 ± 2.487 bc	20.126 ± 3.507 bd	19.79 ± 3.82 bd	18.184 ± 1.128 d

Note: Cows were assigned into either multiparous group (multiparous dairy cows, *n* = 32) or primiparous group (primiparous dairy cows, *n* = 32). The different letters indicate significant differences among treatments, *p* < 0.05.

## Data Availability

The datasets used and analyzed during the current study are available from the corresponding author upon reasonable request.

## Supplementary Materials

The following supporting information can be downloaded at: https://www.mdpi.com/article/10.3390/ani13233693/s1, Figure S1: Atlas of postpartum cervical recovery in parturient cows (5–40 d); Figure S2: Atlas of cervical recovery after parturition in primiparous dairy cows (5–40 d); Figure S3: Atlas of postpartum pregnancy horn recovery in parturient cows (15–40 d); Figure S4: Atlas of postpartum pregnancy horn recovery in primiparous dairy cows (15–40 d); Figure S5: Atlas of postpartum non-pregnant uterine horn recovery in multiparous dairy cows (15–40 d); Figure S6: Atlas of postpartum non-pregnant uterine horn recovery in primiparous dairy cows (15–40 d).
