# Supplementary material for: Study on the Pattern of Postpartum Uterine Involution in Dairy Cows
Source: Animals (Basel). 2023 Nov 29;13(23):3693. doi: 10.3390/ani13233693 (PMC10705072; doi:10.3390/ani13233693)
Supplement: Supplementary file 1 [file animals-13-03693-s001.zip › animals-2680452-supplementary.pdf]

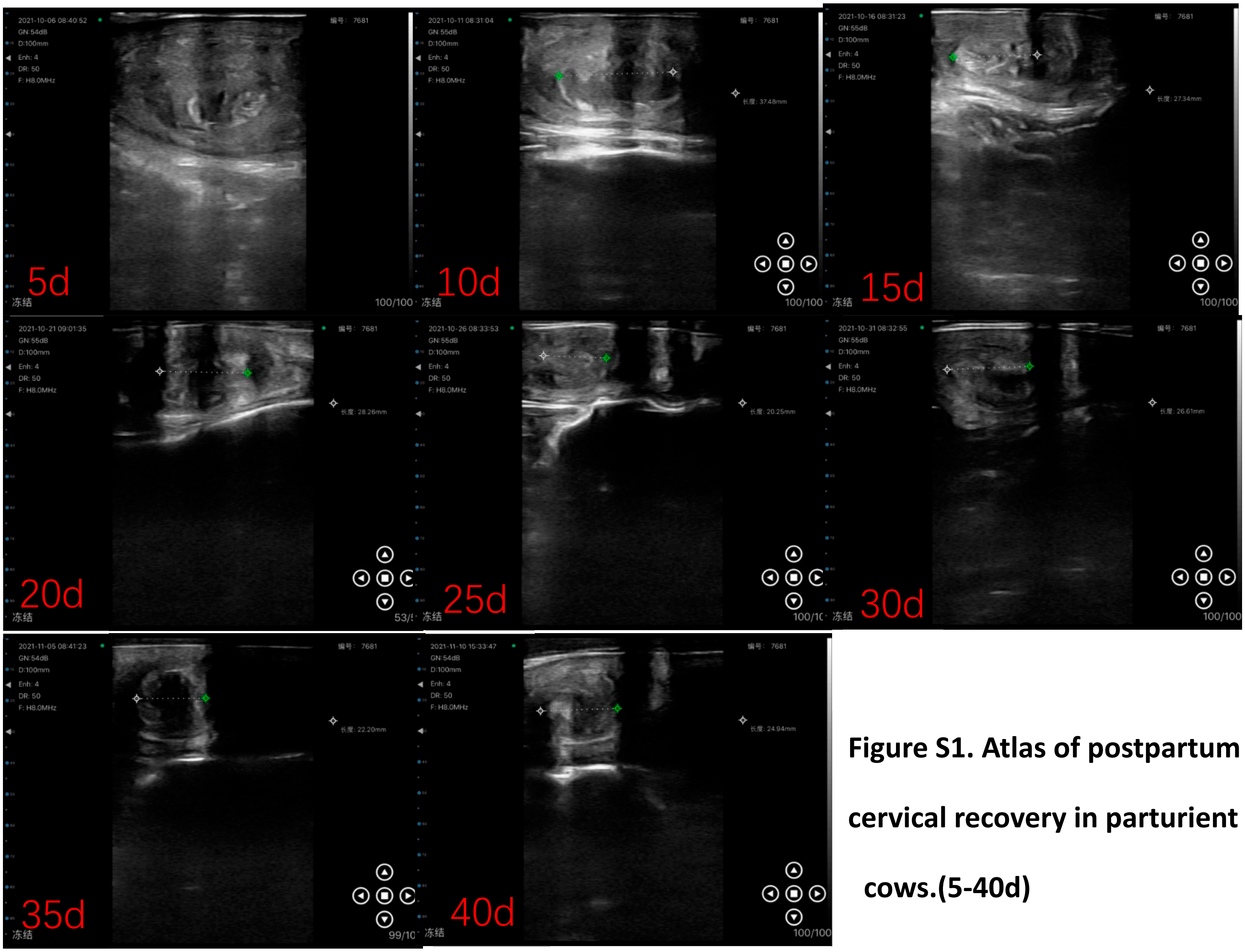

Figure S1. Atlas of postpartum cervical recovery in parturient cows.(5-40d)

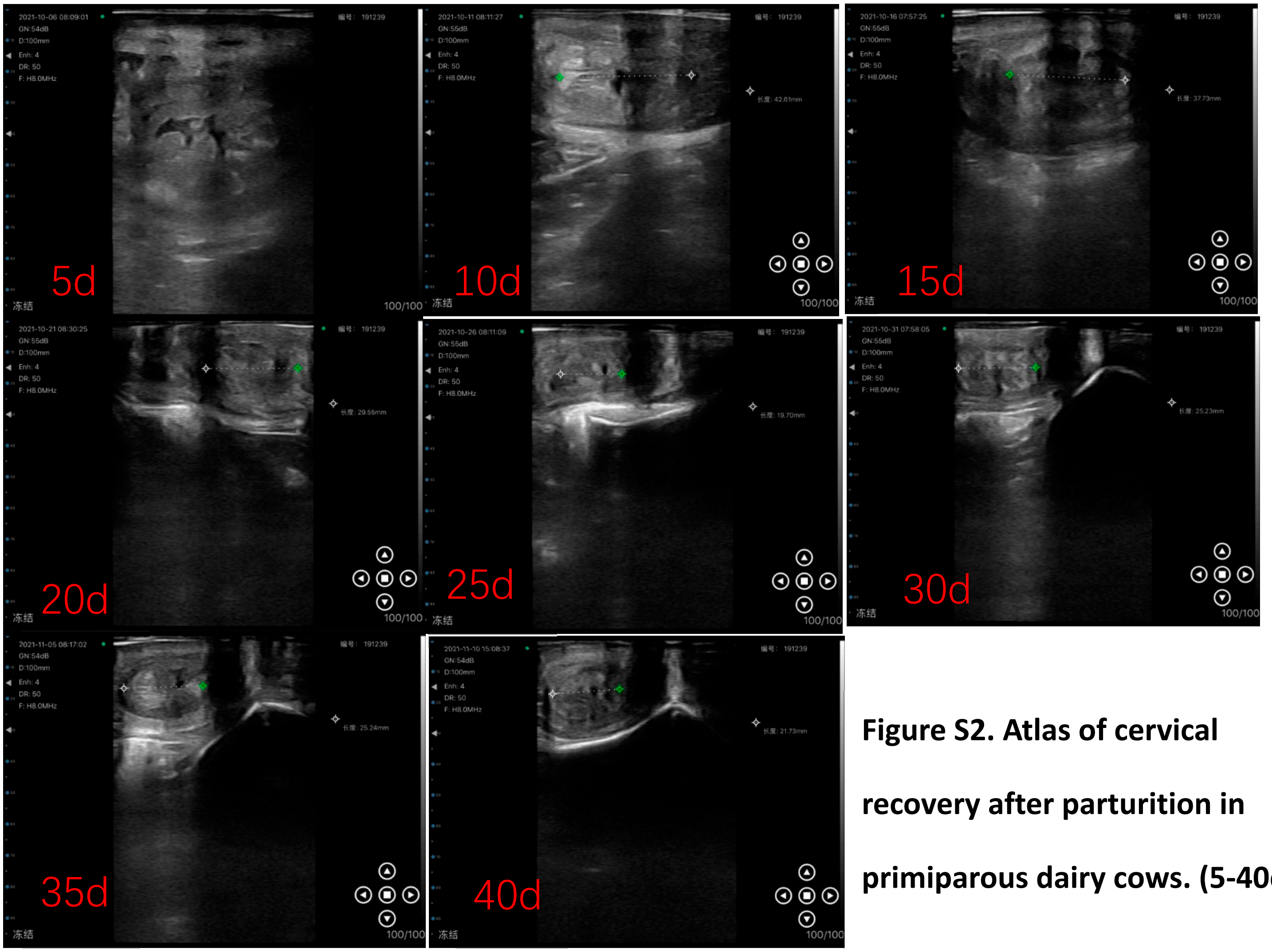

Figure S2. Atlas of cervical recovery after parturition in primiparous dairy cows. (5-40d)

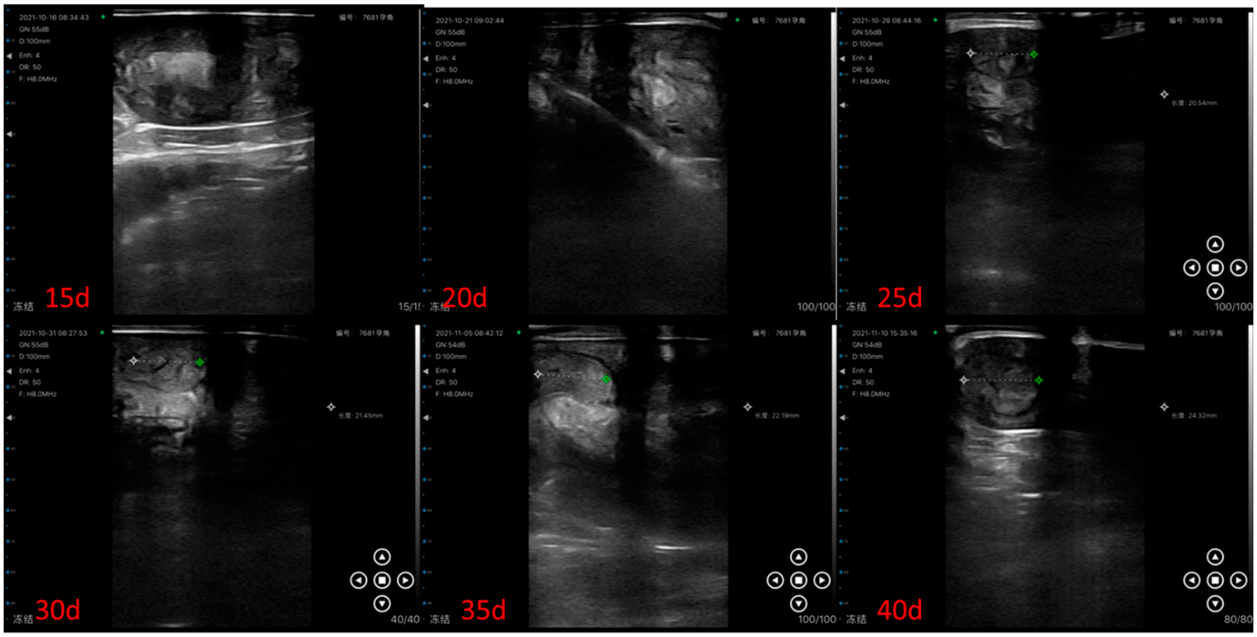

Figure S3. Atlas of postpartum pregnancy horn recovery in parturient cows（15-40d）

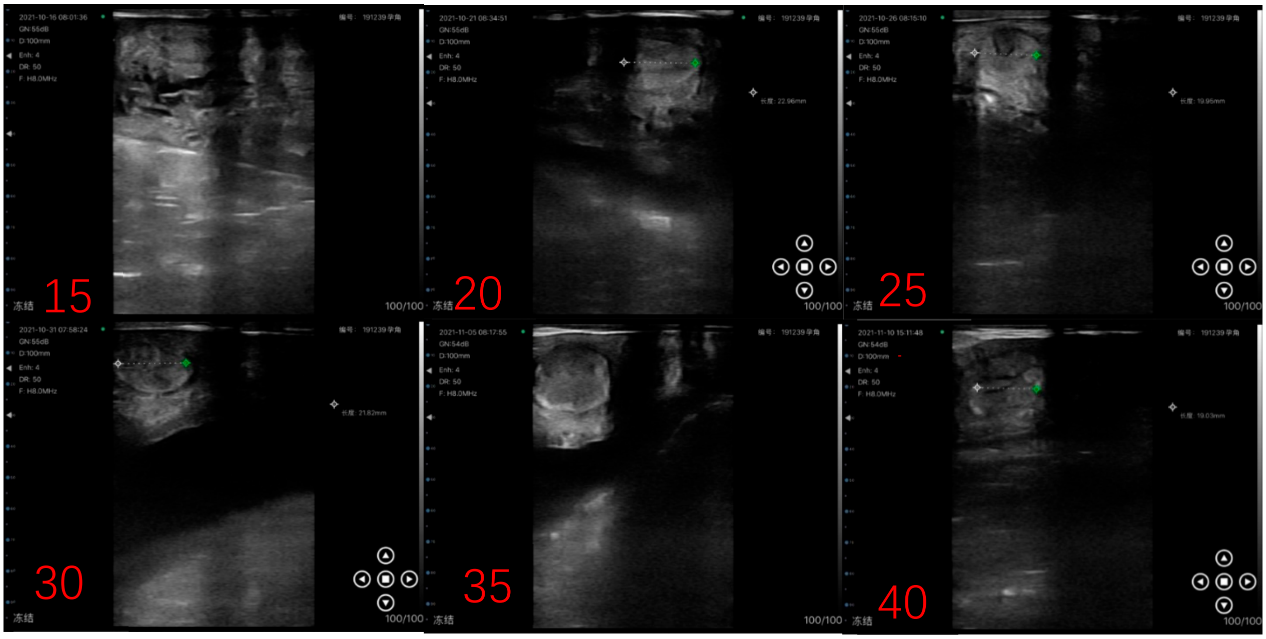

Figure S4: Atlas of postpartum pregnancy horn recovery in primiparous dairy cows.(15-40d)

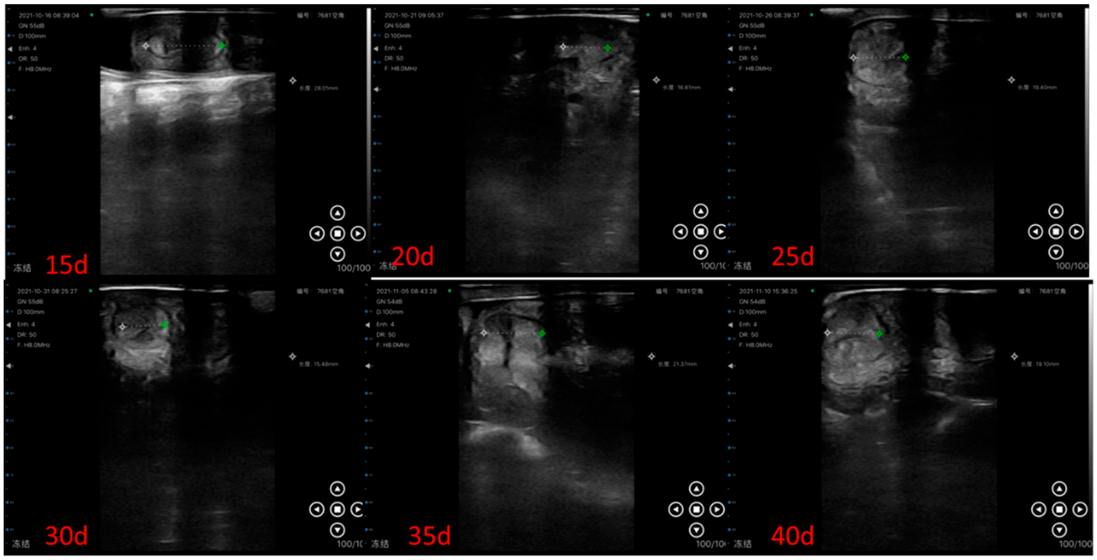

Figure S5. Atlas of postpartum non-pregnant uterine horn recovery in multiparous dairy cows（15d-40d）

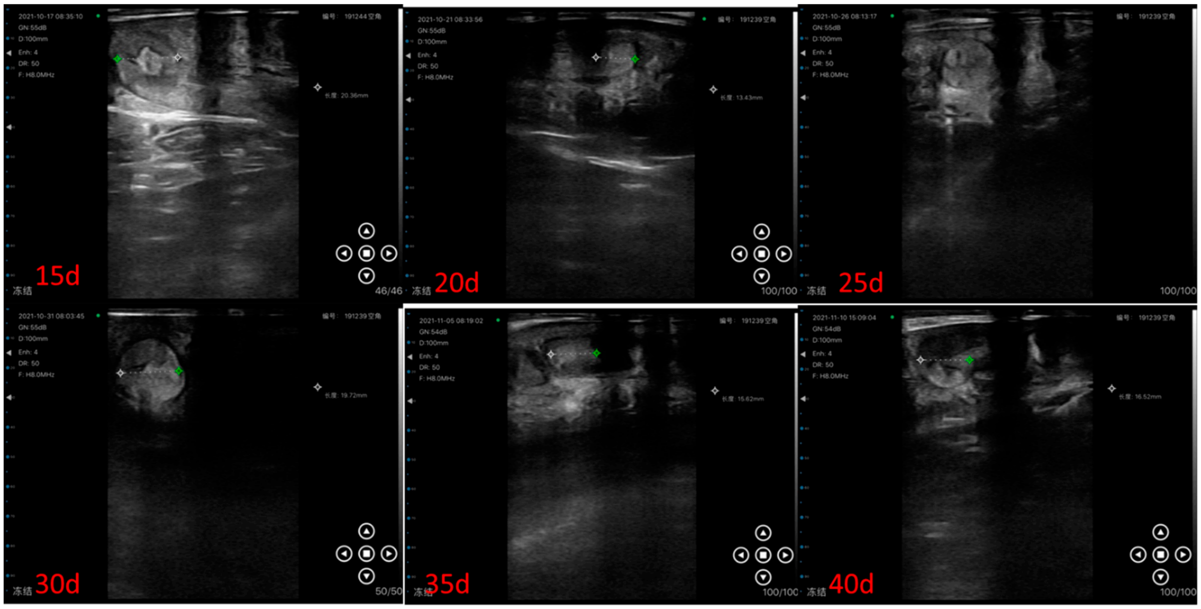

Figure S6. Atlas of postpartum non-pregnant uterine horn recovery in primiparous dairy cows.(15-40d)
